# Household Water Security, Hygienic Handling Practices, and Fecal Contamination: A Mediation Analysis From Wenago District, Gedeo Zone, South Ethiopia

**DOI:** 10.1155/bmri/7129618

**Published:** 2026-04-24

**Authors:** Haimanot Birhanu Beraso, Wagaye Alemu Zenebe, Mekonnen Birhanie Aregu, Negasa Eshete Soboksa

**Affiliations:** ^1^ Weka Dibitu Health Center, Raphe District Health Office, Dilla, Ethiopia; ^2^ School of Public Health, College of Medicine and Health Science, Dilla University, Dilla, Ethiopia, du.edu.et; ^3^ Department of Environmental Health, College of Medicine and Health Science, Dilla University, Dilla, Ethiopia, du.edu.et

**Keywords:** fecal contamination, households water security, water handling practices

## Abstract

Worldwide, over 2 billion people lack access to safe drinking water, often relying on unsafe sources that increase hygiene risks and fecal contamination. This study assessed the mediating role of hygienic water handling practices in the relationship between household water security and fecal contamination in Wenago District, Gedeo Zone, South Ethiopia. A community‐based cross‐sectional survey was conducted between January and April 2024, involving 411 randomly selected households. Data were collected through structured interviews, whereas microbiological analysis of stored water samples was performed using a Wagtech portable testing kit and membrane filtration technique. Statistical analyses were conducted, summarizing descriptive findings in both text and tables. Mediation analysis utilized multiple linear regression models to evaluate the direct and indirect effects. Findings showed that 69.1% of households practiced good water handling, 94.9% faced insecurity, and 93.4% of samples were fecally contaminated. Mediation analysis indicated a significant indirect effect of household water security on contamination through handling practices (*B* = 13.726; 95% CI: 11.400–16.050; *p* < 0.001). The direct effect remained significant (*B* = 28.019, 95% CI: 25.718, 30.321, *p* < 0.001), confirming partial mediation. These results underscore the importance of hygienic handling in reducing contamination risks and safeguarding public health.

## 1. Introduction

Access to clean and safe water is recognized by the United Nations as a fundamental human right, essential for safeguarding public health and ensuring human dignity [1]. Yet, billions of people worldwide continue to lack safely managed drinking water and sanitation services. Marginalized populations, particularly those in low‐income, rural, and periurban settings, remain disproportionately affected due to systemic inequities in water supply planning, investment, and service delivery. Ensuring water that is sufficient, safe, acceptable, physically accessible, and affordable for domestic use remains a major global challenge, especially in regions with inadequate infrastructure and weak governance systems [1–3].

Globally, fecal contamination of drinking water is a critical public health concern. As of 2023, the World Health Organization estimates that 1.7 billion people consume drinking water contaminated with fecal matter, exposing communities to pathogens responsible for diarrhea, cholera, typhoid fever, dysentery, and polio [4]. These infections account for more than half a million diarrheal deaths annually, with children under five disproportionately affected. The Centers for Disease Control and Prevention similarly emphasizes that unsafe water, inadequate sanitation, and poor hygiene facilitate not only enteric infections but also the spread of antimicrobial‐resistant pathogens, intensifying morbidity and mortality risks in low‐resource environments [5].

Household water insecurity, defined by limited availability, accessibility, and reliability, affects both urban and rural populations. It is driven by deficiencies in water distribution systems, treatment technologies, storage facilities, and institutional support structures [2, 3]. Consequently, many households rely on unprotected and unsafe sources, including contaminated surface water, shallow wells, and seasonal streams, which substantially heighten the risk of exposure to waterborne pathogens. Evidence indicates that inadequate water access compromises essential hygiene practices and contributes to the continued transmission of enteric diseases in vulnerable communities [6].

Emerging evidence highlights the significant role of household‐level water storage and handling practices in determining the microbiological quality of drinking water. Studies show that microbial contamination can increase substantially during storage, even when water is initially collected from improved sources [7]. For example, a study in Ethiopia has demonstrated that unsafe handling, prolonged storage, and inadequate cleaning of containers, particularly metal vessels, can markedly elevate bacterial contamination over time, underscoring the need for stronger public health interventions promoting safe household water management [8].

Hand hygiene is a central component of safe water handling. Studies from Ethiopia and other low‐income settings show that handwashing at critical times, such as before handling water or food and after defecation, significantly reduces diarrheal disease and improves child health outcomes [9, 10]. A randomized controlled trial in Ethiopia demonstrated a 41% reduction in diarrhea among children under five when households received soap and hygiene education compared with those following routine practices [10]. Beyond reducing diarrheal disease, improved hand hygiene contributes to lowering parasitic infections and healthcare‐associated infections, illustrating its broad public health benefits [11].

Despite the well‐recognized importance of safe water handling, preventive measures such as washing hands before water collection, using clean and covered narrow‐necked containers, and routinely maintaining storage vessels are inconsistently practiced across many resource‐limited settings [12]. These gaps contribute to the recontamination of drinking water at the household level, even when water originates from improved sources, and significantly heightens the risk of fecal contamination and associated illnesses [13].

Although improved household water security is known to support better hygiene behaviors and reduce microbial contamination, the mechanisms linking water security to drinking water safety remain insufficiently understood. In particular, the mediating role of hygienic water handling practices, encompassing water collection, transport, storage, and point‐of‐use behaviors, has not been well quantified, especially in low‐income rural contexts where structural and behavioral determinants of water quality intersect [14, 15].

To address this critical knowledge gap, the present study examines the mediating effect of hygienic water handling practices on the relationship between household water security and fecal contamination in the Wenago District of the Gedeo Zone, South Ethiopia. Specifically, the study investigates how safe handling behaviors influence drinking water quality at the point of use and evaluates their contribution to reducing health risks associated with fecal contamination in a setting characterized by limited water access and inadequate sanitation infrastructure. By generating empirical evidence, this study is aimed at informing the design of integrated water, sanitation, and hygiene (WASH) interventions that target both structural and behavioral determinants of drinking water safety.

## 2. Methods and Materials

### 2.1. Study Area

**Figure 1:**
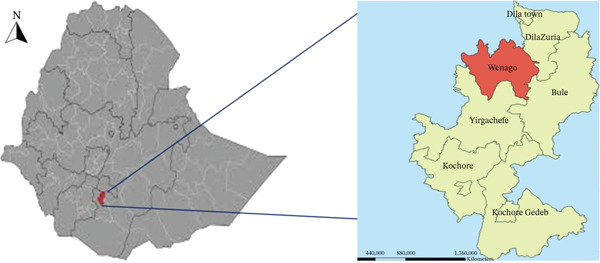
Map of the Wenago District location in the Gedeo Zone, Southern Ethiopia.

The study was conducted in the Wenago District (Figure 1), located in the Gedeo Zone of Southern Ethiopia, approximately 377 km south of Addis Ababa. Geographically, the district extends between 6°12 ^′^30 ^″^ N and 6°22 ^′^00 ^″^ N latitude, and 38°15 ^′^00 ^″^ to 38°20 ^′^00 ^″^ E longitude. According to 2020 projections, Wenago has a population of about 152,000 people and spans an area of 248km^2^, resulting in a high population density of 613 persons per square kilometer, making it one of the most densely populated areas in Ethiopia, with the highest overall fertility rate [16].

Wenago comprises 20 kebeles (smallest administrative structure in Ethiopia), of which 18 are rural and 2 are semiurban. Approximately 90% of the population lives in rural areas, whereas 9.2% live in semiurban areas. The district is bordered by Abaya (Oromia) to the west, Yirgacheffe to the south, Sidama to the north, and Dilla Zuria to the east. The main languages spoken are Gedeuffa, Afan Oromo, and Amharic, with the Gedeo ethnic group being the majority [16].

The study area faces a heightened risk of cholera outbreaks, primarily driven by inadequate hygiene and sanitation infrastructure, limited access to safe drinking water, and high population density [17]. Residents obtain water from a combination of protected and unprotected sources. Water is sourced from both protected and unprotected supplies. Protected sources, such as piped systems, boreholes, and safeguarded wells or springs, are specifically designed to minimize contamination, particularly from fecal matter, and are generally considered safe for consumption. In contrast, unprotected sources, including open wells, springs, and surface water, are classified as unsafe for drinking under the WHO/UNICEF Joint Monitoring Program standards [18]. Despite the availability of protected springs, nearly half of households depend on unprotected streams and rivers. Across all kebeles, rivers, unprotected streams, and rainwater collected during the rainy season serve as supplementary or alternative water sources, further compounding the community′s vulnerability to waterborne diseases.

### 2.2. Study Design and Period

A community‐based cross‐sectional study design was conducted to determine the link of water security status of households to the link between water handling practice and fecal contamination potential in the Wenago District, Gedeo Zone, South Ethiopia, January 01–April 30, 2024.

### 2.3. Study Population

The entire population residing in Wenago District served as the source population for this study. The study population included all households in four randomly selected kebeles during the study period. The study unit consisted of 411 households randomly selected from these kebeles. This sampling approach ensured a representative distribution across the selected areas, enabling a comprehensive analysis and enhancing the generalizability of the findings to the wider district population.

### 2.4. Eligibility Criteria

All household respondents for the study and all household respondents aged greater than or equal to 18, households that lived 6 months and above in the study area were included. If the father was sick, the mother was included, or vice versa, but if both were sick, their child, whose age is greater than 18, was included. Households that were not present during the two‐visit data collecting sessions were not included. Households that were not permanent residents and those unable to respond due to illness were excluded from the survey.

### 2.5. Sample Size Calculation and Procedure

The sample size for this study was calculated using a single population proportion formula, guided by three specific objectives. Each objective required a separate calculation to estimate population proportions with a 5% margin of error at a 95% confidence interval. For household water insecurity, a proportion of 30.8% from a study in Eastern Ethiopia [19] was used, resulting in a sample size of 328. Similarly, for household water handling practices, a proportion of 65.5% from a study in the Chencha District, South Ethiopia [20], yielded a sample size of 347. The third objective, assessing fecal contamination in home‐stored drinking water, was informed by evidence from studies conducted in the Mecha and Fogera Districts of Ethiopia, which reported that 58% of household stored water samples were contaminated with *Escherichia coli* [21]. This prevalence estimate was used to determine the required sample size of 374. Given that the highest calculated sample size was 374, this was selected as the base sample, with an added 10% nonresponse rate (37), bringing the final sample size to 411.

Four kebeles were selected from a total of 20 using a purposive sampling method, specifically chosen based on water accessibility. Subsequently, 411 households were randomly sampled from these kebeles using a simple random sampling technique. Given the wide geographic dispersion of the study population, the sample was proportionally distributed among the selected kebeles according to their population sizes. This approach ensures a fair and accurate representation while addressing the research objectives effectively.

### 2.6. Study Variables

The study examined three core variables: the independent variable (household water security), the dependent variable (fecal contamination of household water), and the mediating variable (hygienic water handling practices).

Household water security, the independent variable, was measured using the 12‐item Household Water InSecurity Experiences (HWISE) Scale, which assesses how frequently households experienced water‐related challenges, such as worry, interruption of supply, reduced ability to wash clothes or hands, disruptions to daily plans or food preparation, inadequate body hygiene, insufficient drinking water, anger, going to sleep thirsty, having no water at all, or feelings of shame, over the previous 4 weeks. Each item is scored from 0 (“never”) to 3 (“often/always”), producing a total score ranging from 0 to 36, with higher scores indicating more severe water insecurity. Based on HWISE validation guidelines, households scoring 0–11 are classified as water secure, whereas those scoring 12–36 are classified as water insecure, a threshold shown to effectively distinguish households experiencing recurrent and meaningful water‐related disruptions [22].

The dependent variable, fecal contamination of household water, measured the level of microbial contamination present in stored or drinking water. Laboratory analysis of home‐stored water samples was used to classify contamination levels. Samples containing 0 CFU/100 mL were considered safe, those with 1–10 CFU/100 mL were classified as low‐risk, samples with 11–100 CFU/100 mL as moderate‐risk, and samples with more than 100 CFU/100 mL as high‐risk fecal contamination [23].

The mediating variable, hygienic water handling practices, was included to explain the mechanism through which household water security may influence fecal contamination. These variables captured behaviors are intended to minimize microbial contamination during the collection, storage, and handling of drinking water. These practices were assessed using 16 items. Participants scoring 8 or more were categorized as having good water handling practices, whereas those scoring fewer than 8 were classified as having poor practices [24].

### 2.7. Data Collection Tool

A data collection tool was developed based on a comprehensive review of the relevant literature [18, 22, 24, 25] and was designed to capture four major domains essential for assessing household water conditions: (1) demographic and socioeconomic characteristics, (2) sanitation status of households, (3) household water‐handling practices, and (4) household water‐security status. It was initially prepared in English, then translated into the local language (Gedeuffa) to enhance clarity and accessibility. To ensure accuracy and consistency, the translated version was subsequently back‐translated by professional linguists.

Demographic and socioeconomic information was collected through a structured questionnaire that included items on age, sex, marital status, ethnicity, religion, educational background, family size, occupational status, and household income. The sanitation status of households was evaluated using indicators such as the type of sanitation facility used, the presence of a handwashing facility near the toilet, routine toilet‐cleaning practices, perceived adequacy of community sanitation facilities, waste‐disposal practices near the main water source, and overall perceptions of community sanitation conditions.

Household water‐handling practices were assessed using a detailed set of variables, including satisfaction with the primary water source, receipt of education on safe water handling, handwashing with soap before collecting water, regular cleaning of storage containers, placing containers in shaded areas, ease of cleaning containers, use of separate containers for drinking and other purposes, adoption of water‐treatment methods, experiences with water‐related illnesses, seasonal changes affecting water supply and quality, daily water‐storage habits, daily water consumption, the presence of community water‐safety initiatives, perceived improvements in water safety over the past year, and the frequency of community discussions regarding water issues.

Household water‐security status was measured by examining the frequency of challenges in accessing clean and safe drinking water, concerns about having enough water for household needs, interruptions or limitations in the main water source, the extent to which water problems hindered household tasks such as washing clothes, changes in daily routines caused by water shortages, modifications in food choices due to water scarcity, lapses in handwashing after unsafe activities, frequency of going without washing the body, experiences of insufficient drinking water, feelings of anger related to water problems, going to sleep thirsty due to limited water availability, incidents of having no usable or drinkable water, and feelings of shame or social exclusion caused by water‐related difficulties. An overall household water‐security score was generated from these indicators to categorize households as either water‐secure or water‐insecure.

### 2.8. Data Collation Procedure Quality Management

#### 2.8.1. Survey Data

Data collection was conducted through face‐to‐face interviews lasting 30–45 min, administered by trained health extension workers. Prior to fieldwork, data collectors underwent a 4‐day training program covering protocols, ethical considerations, and effective communication techniques. To validate clarity and reliability, a pilot test involving 5% of the sample households was carried out. Throughout the process, the principal investigator provided continuous supervision via regular field visits, addressing challenges as they arose. Daily reviews of complete questionnaires ensured completeness and accuracy, with inconsistencies promptly resolved.

Quality assurance measures were integrated at every stage. These included extensive training of data collectors, pilot testing, translation and back‐translation of tools, and systematic supervision. Participants were fully informed about the study′s purpose and their right to withdraw at any time, with verbally informed consent obtained before interviews commenced. Together, these procedures safeguarded the reliability, validity, and ethical integrity of the data collection process.

#### 2.8.2. Water Sample Collection and Analysis

Water samples were collected by trained laboratory technicians using standardized procedures that followed the guidelines set by the WHO [23]. The analyses of these samples were conducted according to the methods described by APHA [26]. Prior to collection, all necessary materials, including sterilized sample bottles, labels, hand sanitizer, soap, and ice packs for transport, were prepared. Consent was obtained from households before sampling, and technicians sanitized their hands before handling the bottles. A total of 411 water samples were collected in sterile 100‐mL plastic bottles, with each bottle labeled using a unique code. The water sample was collected, kept cold during transport to the laboratory, and analyzed within 6 h to maintain integrity.

In the laboratory, strict aseptic techniques, full use of personal protective equipment, and standardized dilution procedures were implemented to ensure the reliability and reproducibility of microbiological analyses. Unique coding of samples, controlled transport temperatures, and carefully monitored incubation conditions further preserved data integrity. Of the total samples, 100 were visibly turbid at the time of collection and were diluted at a 1 : 1 ratio with sterile distilled water prior to processing. This dilution step was quality assured through recovery validation, linearity checks, and the use of procedural blanks to confirm accurate quantification while ruling out dilution‐related bias. Alongside the test samples, a blank control prepared with sterile distilled water was processed, incubated, and analyzed under identical conditions. The blank control served as a contamination check; the absence of colony formation confirmed that media, filtration units, and incubation environments remained free from external contamination throughout the analytical process.

Each 100‐mL sample was filtered through a 0.45‐*μ*m pore‐size membrane filter, which was then placed on absorbent pads in sterile Petri dishes. Membrane Lauryl Sulfate Broth was added, and plates were labeled and allowed to presoak for 4 h before incubation at 44°C for 18 h. Following incubation, plates were equilibrated briefly at room temperature before enumerating yellow colony‐forming units indicative of *E. coli.* For diluted samples, colony counts were multiplied by two to generate the final reported value. The integration of procedural blanks, blank controls, and validated dilution steps ensured high analytical accuracy and minimized the risk of contamination or measurement error across all microbiological assessments.

### 2.9. Data Analysis/Simple Mediational Analysis

Data were entered, coded, and cleaned, and then analyzed using SPSS Version 25.0. Descriptive statistics were first computed to summarize sociodemographic characteristics and key study variables. Categorical variables were presented using frequency tables and bar graphs, whereas continuous variables were summarized using means and standard deviations. Normality was assessed using the Shapiro–Wilk test. Where parametric assumptions were met, standard analyses were performed; otherwise, robust or bootstrapped estimators were used. Additionally, variance inflation factor (VIF) values were calculated to detect multicollinearity among independent variables, confirming that multicollinearity was not a concern.

Mediation analysis was conducted using Andrew Hayes′ PROCESS macro (Model 4), a widely applied and efficient framework for estimating indirect effects through multiple linear regression. This analytical approach was selected to clarify the mechanism through which household water security influences fecal contamination in Wenago District. Although water security may exert a direct effect on microbial contamination, its influence frequently operates through behavioral pathways—particularly hygienic water‐handling practices such as safe storage, hygienic transfer, and protection against recontamination. These behaviors form the critical link between water conditions at the source and water quality at the point of use.

**Figure 2:**
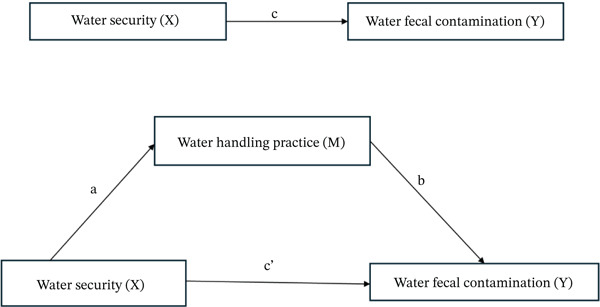
Conceptual mediation model linking household water security (*X*), hygienic water‐handling practices (*M*), and fecal contamination (*Y*).

Within this framework, Figure 2 illustrates household water security (*X*) as the predictor, hygienic handling practices (*M*) as the mediator, and fecal contamination (*Y*) as the outcome. We first estimated the total effect of water security on fecal contamination (Path c). The mediator was then introduced to decompose this association into the direct effect of water security on contamination (Path c ^′^) and the indirect effect transmitted through handling practices (Paths a and b). The indirect effect (ab), the primary indicator of mediation, was calculated using the product‐of‐coefficients method with bootstrap confidence intervals to strengthen statistical inference.

Analytically, water security was regressed on hygienic handling practices to estimate Path a, followed by regressing fecal contamination on the mediator to estimate Path b. Both variables were then entered simultaneously to obtain the adjusted direct effect (c ^′^). Bootstrap‐derived confidence intervals allowed for robust testing of the indirect pathway without assuming normality. To enhance interpretability, both standardized and unstandardized coefficients were reported.

### 2.10. Ethical Consideration

Ethical approval was secured from the Institutional Review Board of Dilla University College of Medicine and Health Sciences. Prior to the commencement of data collection, participants were provided with a brief overview of the study′s purpose. They were informed that their participation was entirely voluntary, that they had the right to withdraw from the study at any time, and about their responsibilities during the data collection process. Participants were informed about the purpose of the study, and verbal consent was obtained from everyone. All personal information was kept confidential, and the collected data were used solely for the intended research purposes.

## 3. Result

### 3.1. Sociodemographic Characteristics

The mean age of the participant was 31.68 (SD = 13.14) of this the majority (85.6%) were aged between 20 and 40 years, whereas 4.6% (19/411) were between 61 and 80 years. In this study, females were about 59.4% more than males (40.6%), around 90% of the participant were married. Majority of the participants were orthodox, nearly half of participants had completed primary education (53.3%), and their occupation were merchants (50.4%). 40.6% of participants′ monthly income was less or equal to ETB1000 (Ethiopian Birr), households having 2 up to 4 family members accounts for 68.6% (Table 1).

**Table 1:** Sociodemographic characteristics of participants (*N* = 411).

Socio demographic variable	Categories	Frequency	Percentage (%)
Sex	Males	167	40.6
	Female	244	59.4
Age	20–40	352	85.6
	41–60	40	9.7
	61–80	19	4.6
Marital status	Married	373	90.8
	Divorced	38	9.2
Educational status	Illiterate	102	24.8
	Primary level	219	53.3
	Secondary level	77	18.7
	College/university	13	3.2
Occupation	Unemployed	77	18.7
	Merchant	207	50.4
	Farmer	77	18.7
	Daily laborer	50	12.2
Family size	2–4	282	68.6
	5–7	103	25.1
	Above 7	26	6.3
Income (ETB)	≤ 1000	167	40.6
	1000–1500	65	15.8
	1500–2000	117	28.5
	2000–2500	62	15.1

### 3.2. Sanitation Status of Households

Table 2 presents the sanitation conditions of the study households. None of the households surveyed have access to a flush toilet connected to a sewage system; instead, all rely on traditional pit latrines. A large proportion (68.1%) lacks a handwashing facility near their toilet, with only 31.9% having such a facility available. Nevertheless, all respondents reported that they clean their toilet facilities regularly.

**Table 2:** Sanitation status of households in Wenago District, Gedeo Zone (*N* = 411).

Variables	Frequency		Percentage (%)
Access to a flush toilet connected to a sewerage system.	No	411	100
Access to a traditional pit latrine.	Yes	411	100
Toilets with hand washing facility.	No	280	68.1
	Yes	131	31.9
Regular toilet facility cleaning practice.	Yes	411	100
Community′s belief towards adequacy of sanitation facilities.	No	411	100
Waste disposal by the community near to water sources.	No	180	43.8
	Yes	231	56.2
Are you concerned about the impact of waste disposal on drinking water quality?	No	120	29.2
	Yes	291	70.8
Do you think that cultural beliefs affect how your household addresses it?	No	74	18.0
	Yes	337	82.0
Do you believe that cultural practices can result in fecal contamination of stored drinking water in your home?	No	119	29.0
	Yes	292	71.0

Every respondent (100%) believes that the community′s sanitation facilities are inadequate. More than half of respondents (56.2%) reported that waste was being disposed of near their main water source. A significant majority (70.8%) have experienced problems with the quality of their drinking water. Additionally, a large proportion (82.0%) believes that cultural beliefs influence how their households manage water, and most (71.0%) think that cultural practices may contribute to fecal contamination in stored drinking water.

### 3.3. Water Security Status of Households

The household survey about their experiences with water security over the past 4 weeks revealed that a significant majority (60.1%) of respondents reported often or always facing challenges in accessing clean and safe drinking water. Although most respondents (61.8%) never worried about having enough water for household needs, a notable 24.1% reported frequent concerns. More than half (54.5%) experienced regular interruptions or limitations in their main water source, and approximately 24.1% showed that water problems often or always prevented them from washing clothes.

More than half of the respondents (55.5%) had to change their schedules or plans due to water issues, and a smaller percentage (20.2%) had to alter what they ate because of water problems. A significant 55.7% reported often or always going without washing their hands after unsafe activities, whereas 59.4% never went without washing their body, though 29.2% did so sometimes. Nearly half (45.7%) showed that there was often or always not enough water to drink, and a substantial 28.2% felt angry about their water situation often. Almost half (49.4%) went to sleep thirsty sometimes due to insufficient water, and about 23.8% reported often or always having no usable or drinkable water. A significant number (39.9%) felt ashamed or excluded from sometimes due to water issues. Of the 411 participants, 21 households (5.1%) were water‐secure, whereas the remainder were water‐insecure (Table 3).

**Table 3:** Water security status of households Wenago District (*N* = 411).

Variables	Categories, frequencies, and percentage							
	Never		Rarely		Sometime		Often/always	
	No	%	No	%	No	%	No	%
In the last 4 weeks, how frequently have you or anyone in your household faced challenges in accessing clean and safe drinking water at home?	63	15.3	1	0.2	100	24.3	247	60.1
In the last 4 weeks, how frequently have you or anyone in your household worry you would not have enough water for all of your household needs?	254	61.8	5	1.2	53	12.9	99	24.1
In the last 4 weeks, how frequently has your main water source been interrupted or limited (e.g., water pressure, less water than expected, and river dried up)?	87	21.2	14	3.4	86	20.9	224	54.5
In the last 4 weeks, how frequently have you or anyone in your household had to change schedules or plans due to problems with your water situation?	51	12.4	5	1.2	127	30.9	228	55.5
In the last 4 weeks, how frequently have you or anyone in your household had to change what was being eaten because there were problems with water?	272	66.2	4	1.0	83	20.2	52	12.7
In the last 4 weeks, how frequently have you or anyone in your household had to go without washing their hands after dirty activities?	62	15.1	9	2.2	111	27.0	229	55.7
In the last 4 weeks, how frequently have you or anyone in your household had to go without washing their body because of problems with water?	244	59.4	8	1.9	120	29.2	39	9.5
In the last 4 weeks, how frequently has there not been as much water to drink as you would like to you or anyone in your household?	138	33.6	1	0.2	84	20.4	188	45.7
In the last 4 weeks, how frequently did you or anyone in your household feel angry about your water situation?	212	51.6	0	0	83	20.2	116	28.2
In the last 4 weeks, how frequently have you or anyone in your household gone to sleep thirsty because there wasn’t any water to drink?	112	27.3	3	0.7	203	49.49	3	22.6
In the last 4 weeks, how frequently has there been no useable or drinkable water whatsoever in your household?	148	36.0	56	13.6	109	26.5	98	23.8
In the last 4 weeks, how frequently have problems with water caused you or anyone in your household to feel ashamed/excluded/stigmatized?	131	31.9	1	0.2	164	39.9	115	28.0
Water secured household 21 (5.1).								
Water insecure household 390 (94.9).								

### 3.4. Water Handling Practices of Households

A household survey on water handling practices revealed several important findings. A significant portion of respondents (58.4%) expressed dissatisfaction with their primary water source. Additionally, 56.2% reported not having received effective education on safe water handling practices. Although 54.0% of households practice handwashing with soap before collecting water, container hygiene habits were more evenly split, with 49.6% regularly cleaning their containers and 51.6% keeping them in shaded areas. About 57.2% of participants found their containers easy to clean. Furthermore, 69.3% of households used separate containers for drinking and other purposes, and 68.6% practiced some form of water treatment before consumption.

Despite these efforts, 60.1% of households reported experiencing water‐related illnesses. Seasonal changes were also a concern, with 54.0% noting that such variations affected their water supply and quality. In terms of water usage, 52.8% stored water for daily use, and 54.5% consumed less than 100 L of water per day. Community‐level engagement appeared limited, as 56.0% of respondents showed there were no initiatives focused on improving water safety, 61.3% saw no improvements in the past year, and 63.7% said that water issues were not regularly discussed within their communities (Table 4).

**Table 4:** Water handling practice of households in Wenago District (*N* = 411).

Variable	Category	Frequency	Percentage (%)
Household satisfaction with its primary water source.	Yes	171	41.6
	No	240	58.4
Households received effective training on safe water handling practice.	Yes	180	43.8
	No	231	56.2
Households practice hand washing with soap before collecting water.	Yes	222	54.0
	No	189	46.0
Regular cleaning of water storage containers.	No	207	50.4
	Yes	204	49.6
Keeping water storage container in shaded area.	Yes	212	51.6
	No	199	48.4
Does the container used for collecting drinking water is comfortable for easily washing? If it is bucket and clay pot, answer yes; if other than this, answer no.	Yes	235	57.2
	No	176	42.8
Regular and proper coverage of water container.	Yes	271	65.9
	No	140	34.1
Is water taken from the drinking water container by pouring (yes)/dipping (no)?	Yes	285	69.3
	No	126	30.7
Separate water containers for drinking and other purpose.	Yes	282	68.6
	No	129	31.4
Using homemade water treatment methods before consuming such as boiling, chlorination, filtering, and solar disinfection.	Yes	247	60.1
	No	164	39.9
Anyone of the family members who has experienced a water related illness?	Yes	222	54.0
	No	189	46.0
Seasonal changes that affect water supply and water quality.	Yes	217	52.8
	No	194	47.2
Households store water for daily use.	Yes	187	45.5
	No	224	54.5
Households with daily water consumption of more than 100 L.	Yes	181	44.0
	No	230	56.0
Community initiatives focused on improving water safety	Yes	159	38.7
	No	252	61.3
Have you seen improvement in local water supply management in the last 1 year?	Yes	149	36.3
	No	262	63.7
Water handling practice	Poor	127	30.9
	Good	284	69.1

### 3.5. Fecal Contamination of Drinking Water Stored at Home

Laboratory analysis of home‐stored drinking water showed that only two households (0.5%) had water that was free from fecal contamination (0 CFU/100 mL), which is considered safe. Twenty‐five households (6.1%) had a low level of fecal contamination (1–10 CFU/100 mL), whereas the vast majority, 384 households (93.4%), had moderate fecal contamination (11–100 CFU/100 mL). No household water samples showed contamination levels above 100 CFU/100 mL, the threshold for high‐risk contamination.

### 3.6. Mediation Analysis Results

**Figure 3:**
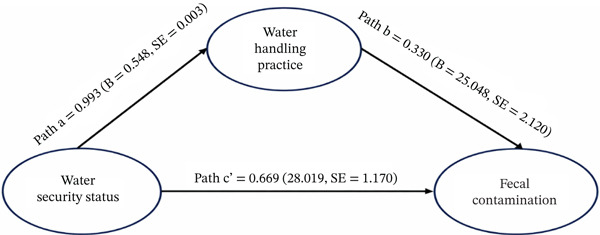
Path model of household water security, handling practices, and fecal contamination risk in stored drinking water.

Table 5 presents the results of the regression analysis. The mediation analysis demonstrated that household water security was strongly and significantly associated with hygienic water‐handling practices (Path a), with an unstandardized coefficient of *B* = 0.548 (SE = 0.003, *p* < 0.001), and a 95% confidence interval ranging from 0.542 to 0.555. In turn, hygienic handling practices were a significant predictor of fecal contamination levels (Path b), showing a sizeable effect (*B* = 25.048, SE = 2.120, *p* < 0.001), with a 95% CI of 20.879 to 28.217. When both water security and handling practices were included in the model, the direct effect of water security on fecal contamination (Path c ^′^) remained strong and statistically significant (*B* = 28.019, SE = 1.170, *p* < 0.001; 95% CI: 25.718–30.321), indicating that hygienic handling partially mediated this relationship (Figure 3).

**Table 5:** Association between water handling practice, water security status and fecal contamination potential of home stored drinking water.

Relationship	Unstandardized Beta (*B*)	*t*‐stat	Standard error	*p*value	95% CI	Standardized coefficients (*β*)
The relationships of household water security status and water handling practice (*Path a*)	0.548	166.757	0.003	0.001	0.542, 0.555	0.993
The relationship between water handling practice of households and fecal contamination of home stored drinking water in the presence water security (*Path b*)	25.048	11.812	2.120	0.001	20.879, 28.217	0.330
The relationships of household′s water security status and fecal contamination of home stored drinking water in the presence water handling practice (Path c ^′^)	28.019	23.931	1.170	0.001	25.718, 30.321	0.669
The overall relationship between household water security and fecal contamination of home stored drinking water (total effect)	41.749	255.974	0.163	0.001	38.510, 44.980	0.997

The estimated indirect effect (*a* × *b*) was *B* = 13.726, representing the portion of the effect transmitted through hygienic handling practices. Bootstrapping with 5000 bias‐corrected samples confirmed that this indirect pathway was statistically significant, with a 95% bootstrapped CI of 11.400–16.050, indicating that the mediating effect was robust and not attributable to sampling variability. The total effect of household water security on fecal contamination (Path c), calculated by combining the direct and indirect components, was *B* = 41.745, with an approximated SE of 1.651 and a 95% CI ranging from 38.510 to 44.980; this aligns with the large standardized total effect previously reported (*β* = 0.997). Overall, the results provide strong evidence that hygienic water‐handling practices substantially mediate, but do not eliminate, the influence of household water security on fecal contamination, supporting a model of partial mediation (Table 6).

**Table 6:** Mediation of water handling practices on the relationship between water security and fecal contamination in stored water.

Relationship	Total effect	Direct effect	Indirect effect	95% CI	*t*‐statics	Conclusion
$\begin{array}{c} Water\;security>\\ water\;handling\;practice>\\ fecal\;contamination \end{array}$	41.749 ^∗^	28.019 ^∗^	13.726 ^∗^	38.510, 44.980	255.974	Partial mediation

^∗^Statistically significant at 95% CI.

## 4. Discussion

This study is aimed at assessing the role of hygienic water handling practices in mediating household water security and reducing fecal contamination of home‐stored drinking water in Wenago District, Gedeo Zone, South Ethiopia. Access to clean, safe, and affordable water for drinking, hygiene, and sanitation is fundamental to human health and overall quality of life, as water insecurity often forces households to consume contaminated water or compromise hygiene practices [27]. In this study, 94.9% of households experienced water insecurity, a proportion substantially higher than the 70% reported across 28 sites in low‐ and middle‐income countries involving 7603 households measured using an 11‐item scale [28], and significantly greater than the 56% reported in a multiple‐mediation study of 1543 households in Eastern Hararghe, Oromia, Eastern Ethiopia [19]. This pronounced disparity underscores the unique environmental and infrastructural challenges in the present study area, including its water topography, limited water infrastructure, and inconsistent water service delivery. The implications of such high levels of water insecurity are far‐reaching, as inadequate access can compel households to utilize unsafe water sources, thereby elevating the risk of waterborne illnesses such as cholera, dysentery, and other enteric infections [3].

Improved handling practices lead to a more secure and dependable water supply, according to the current study, which shows a significant positive relationship between household water handling practices and water security status. This result is consistent with studies carried out in rural Kenya, where community‐based initiatives to enhance water handling and storage were linked to increased household water security and decreased dependence on contaminated water sources [29]. Furthermore, our findings show a significant negative correlation between the potential for fecal contamination and the state of water security. This implies that fecal contamination of stored drinking water is less likely to occur in households with greater water security. Gunda et al., which examine the positive relationship between water quality and water security, supports the current study [30]. The direct effect of water security status on the potential for fecal contamination was still significant but decreased when water handling practice was added as a mediating variable. This suggests water handling plays a key role in the relationship between water security and fecal contamination, influencing it both directly and indirectly. This result is supported by an earlier study, which found that contamination can occur directly via poor hygiene and handling and indirectly through environmental exposure, broken pipelines, or inadequate treatment systems [31, 12].

Water handling practices are crucial in figuring out water quality, health outcomes, and resource management. Effective household water handling is essential for ensuring safe drinking water and improving public health [32]. The findings of this study show that 69.1% of households practiced good water handling. Although encouraging, this finding is slightly lower than the 71.2% reported in a similar cross‐sectional study in Boloso Sore District, Wolaita Zone, Ethiopia [24]. In contrast, a study conducted in Tiyo District, Arsi Zone, revealed a significantly lower rate, with only 53.3% of households practicing proper water handling [33]. The disparity in proper water handling practices highlights geographic variations across Ethiopia. These differences likely stem from factors such as local awareness campaigns, water source accessibility, and sociodemographic variations.

In this study, only 43.8% of respondents demonstrated adequate awareness of safe drinking water handling, a proportion markedly lower than levels reported in other Ethiopian settings, including 65.5% in Chencha District [20], approximately 62% practicing safe handling in Boloso Sore Woreda [24], and 53.3% in Tiyo District, Arsi Zone [33]. These regional discrepancies may reflect differences in the reach and intensity of public health education, access to WASH‐related information, community literacy, and the degree of local engagement in sanitation initiatives. The substantially lower awareness observed in the current study signals a critical knowledge and practice gap, underscoring the need for strengthened, context‐specific health promotion strategies, including targeted community education and behavior‐change communication. Enhancing these interventions is essential to improving safe household water handling and reducing the burden of waterborne diseases.

The percentage of participants who adequately covered their water storage containers to prevent contamination was 65.9%, which was slightly lower than the 67% found in a study conducted in Northern India [34]. This minor discrepancy could result from differences in public health messaging about safe storage, cultural customs, or infrastructure. These results suggest that there is a need for improvement, even though covering containers is an important practice for preserving water quality. Water safety could be improved by increased community education that emphasizes safe storage, especially in places where access to treated water is limited.

A notable concern has arisen on homemade water treatment practices, with 39.9% of households reporting no use of any treatment methods. This is significantly lower than the 74.6% of households in Kenya who also did not treat their water [35]. This disparity highlights significant differences in water handling practices between the two countries, which can be attributed to various factors, including access to resources, public health education, and socioeconomic conditions.

The study also revealed that 65.9% of participants used the pouring method to withdraw water from storage containers, and 49.6% reported regular washing and cleaning of their containers. These findings are consistent with similar studies conducted in Northwest Ethiopia, which also reported 73.0% using the pouring method and regular container cleaning [36]. The consistency in these practices across different regions suggests that certain water handling behaviors are widespread and may be influenced by common factors such as access to clean water, sanitation infrastructure, and public health education.

Fecal contamination of home‐stored drinking water represents a major public health threat, particularly in settings where sanitation systems and water treatment infrastructure are inadequate. Such contamination occurs when water is exposed to fecal matter containing pathogenic bacteria, viruses, and parasites that can cause serious enteric infections [37]. In our study, 93.4% of household drinking‐water samples were contaminated with fecal indicators, suggesting that most households consume water that falls within a moderate‐risk to high‐risk contamination category. This finding aligns with a multicountry cross‐sectional analysis across 27 datasets from low‐ and middle‐income countries (2014–2020), which reported fecal contamination at the point of use ranging between 19% and 99% [38]. However, the level observed in our study is higher than reports from several other regions, including 82.1% in Wegera District, Northwest Ethiopia [39], Nepal of 72% [40] and 58% in Mecha and Fogera Districts of Ethiopia [41]. The results of this study show that the risk of fecal contamination in drinking water stored at home is significantly and favorably correlated with household water handling practices. In particular, the statistical analysis shows a strong correlation between increased risks of fecal contamination and inadequate or poor water handling behaviors. This implies that the microbiological quality of drinking water may be compromised in many households due to inadequate hygienic practices during collection, storage, or retrieval.

These findings are in line with earlier cross‐sectional studies carried out in several locations, such as Boloso Sore District [24], and Dessie [32] in Ethiopia, as well as in Kenya water [35] and Peru [42]. Similar trends were seen in each of these situations, where poor household water management greatly accelerated the decline of water quality, especially regarding fecal contamination. To protect household water safety, this emphasizes the urgent need for focused public health interventions and community education initiatives targeted at enhancing water handling practices.

This study offers several important strengths, particularly its integrated analytical framework, which moves beyond examining fecal contamination, water security, and water handling practices as isolated factors. By exploring the interconnected pathways among these variables, the study provides a more holistic understanding of how household behaviors influence drinking‐water quality. Notably, identifying water‐handling practices as a mediating factor between water insecurity and fecal contamination offers valuable insight into how contamination risks can be mitigated at multiple levels. This finding underscores that households experiencing higher water insecurity are more likely to adopt unsafe handling behaviors, which in turn elevates the risk of microbial contamination.

The practical implications of these results are substantial. The partial mediation effect indicates that interventions addressing both water security (such as improving infrastructure and access) and behavioral components (such as promoting safe handling and storage) are likely to be more effective than focusing on either dimension alone. This dual‐targeted approach is particularly relevant in resource‐limited settings where infrastructure improvements may take time to implement, but behavior‐change interventions can offer more immediate benefits. Additionally, the findings reinforce the need for integrated public health strategies that combine behavioral education, community engagement, and incremental infrastructure upgrades to enhance household water safety. Together, these insights contribute to a more actionable understanding of how to prevent fecal contamination and improve water quality at the point of use.

The study has several limitations. Although sociodemographic characteristics and sanitation conditions were recognized as important covariates, they were not examined through detailed sequential analysis, which may influence the interpretation of observed associations. Reliance on self‐reported data for household characteristics and water handling behaviors introduces potential recall and social desirability biases, such as overreporting of good practices, which may attenuate or inflate the mediation effect. In addition, the cross‐sectional design limits causal inference in the mediation analysis, as the temporal sequence implied, water security affecting handling practices and subsequently contamination, cannot be definitively established from data collected at a single time point. Finally, the applicability of the findings may be limited in contexts with different environmental, cultural, or infrastructural conditions. Despite these constraints, the study highlights the need for integrated approaches that address both behavioral and structural determinants of safe household water use.

## 5. Conclusions

Households employing effective water handling practices, such as safe storage, routine cleaning of containers, and consistent use of appropriate point‐of‐use treatment methods, were found to have significantly lower levels of fecal contamination in their drinking water. These behaviors also contribute to improved water security by ensuring more reliable access to safe water at the household level. The analysis further demonstrates that water handling practices partially mediate the relationship between water security and fecal contamination, indicating that enhancing water security not only directly reduces contamination risks but also promotes safer handling behaviors, which in turn decreases microbial contamination. This partial mediation underscores the importance of addressing both behavioral and structural determinants in efforts to improve household water safety. Accordingly, the findings support the need for comprehensive, multilevel interventions that simultaneously strengthen behavior change and infrastructure. Practical strategies include improving community health worker training to deliver tailored education on safe container hygiene and treatment methods; scaling up point‐of‐use water treatment promotion, such as chlorination, filtration, or solar disinfection, through accessible community initiatives; and increasing investment in water infrastructure to expand protected water sources and improve reliability of supply. By integrating these behavioral, educational, and infrastructural approaches, communities can substantially reduce the risk of fecal contamination and enhance overall safety, reliability, and quality of drinking water.

**Nomenclature**
APHA: American Public Health Association
BCC: behavioral change communication
CI: confidence interval
CLTS: community led total sanitation
DV: dependent variable
HEW: health extension worker
IDP: internal displaced people
LDC: least developed countries
MDG: Millenium Development Goal
RFA: risk factor attribution
SDG: Sustainable Development Goal
SE: Southern Ethiopia
SIDS: Small Island Developing State,
PSS: Statistical Package for the Social Sciences
TNTC: too numerous to count
UNICEF: United Nations Children′s Fund
VIF: variance inflation factor
WASH: water sanitation and hygiene
WHO: World Health Organization

## Funding

No funding was received for this manuscript.

## Disclosure

The authors have nothing to report.

## Conflicts of Interest

The authors declare no conflicts of interest.

## Data Availability

The corresponding author can provide reasonable data requests, calibration records, and details.
